# Real-time fMRI using multi-band echo-volumar imaging with millimeter spatial resolution and sub-second temporal resolution at 3 tesla

**DOI:** 10.3389/fnins.2025.1543206

**Published:** 2025-03-12

**Authors:** Stefan Posse, Sudhir Ramanna, Steen Moeller, Kishore Vakamudi, Ricardo Otazo, Bruno Sa de La Rocque Guimaraes, Michael Mullen, Essa Yacoub

**Affiliations:** ^1^Department of Neurology, University of New Mexico, Albuquerque, NM, United States; ^2^Department of Physics and Astronomy, University of New Mexico, Albuquerque, NM, United States; ^3^Department of Radiology, Center for Magnetic Resonance Research, University of Minnesota, Minneapolis, MN, United States; ^4^Department of Medical Physics, Memorial Sloan Kettering Cancer Center, New York, NY, United States; ^5^Department of Radiology, Memorial Sloan Kettering Cancer Center, New York, NY, United States; ^6^Department of Nuclear Engineering, University of New Mexico, Albuquerque, NM, United States

**Keywords:** echo-volumar imaging, simultaneous multi slab encoding, multi-band encoding, functional MRI, resting-state connectivity, task-based activation, NORDIC denoising, compressed sensing

## Abstract

**Purpose:**

In this study we develop undersampled echo-volumar imaging (EVI) using multi-band/simultaneous multi-slab encoding in conjunction with multi-shot slab-segmentation to accelerate 3D encoding and to reduce the duration of EVI encoding within slabs. This approach combines the sampling efficiency of single-shot 3D encoding with the sensitivity advantage of multi-echo acquisition. We describe the pulse sequence development and characterize the spatial–temporal resolution limits and BOLD sensitivity of this approach for high-speed task-based and resting-state fMRI at 3 T. We study the feasibility of further acceleration using compressed sensing (CS) and assess compatibility with NORDIC denoising.

**Methods:**

Multi-band echo volumar imaging (MB-EVI) combines multi-band encoding of up to 6 slabs with CAIPI shifting, accelerated EVI encoding within slabs using up to 4-fold GRAPPA accelerations, 2-shot k_z_-segmentation and partial Fourier acquisitions along the two phase-encoding dimensions. Task-based and resting-state fMRI at 3 Tesla was performed across a range of voxel sizes (between 1 and 3 mm isotropic), repetition times (118–650 ms), and number of slabs (up to 12). MB-EVI was compared with multi-slab EVI (MS-EVI) and multi-band-EPI (MB-EPI).

**Results:**

Image quality and temporal SNR of MB-EVI was comparable to MS-EVI when using 2–3 mm spatial resolution. High sensitivity for mapping task-based activation and resting-state connectivity at short TR was measured. Online deconvolution of T_2_* signal decay markedly reduced spatial blurring and improved image contrast. The high temporal resolution of MB-EVI enabled sensitive mapping of high-frequency resting-state connectivity above 0.3 Hz with 3 mm isotropic voxel size (TR: 163 ms). Detection of task-based activation with 1 mm isotropic voxel size was feasible in scan times as short as 1 min 13 s. Compressed sensing with up to 2.4-fold retrospective undersampling showed negligible loss in image quality and moderate region-specific losses in BOLD sensitivity. NORDIC denoising significantly enhanced fMRI sensitivity without introducing image blurring.

**Conclusion:**

Combining MS-EVI with multi-band encoding enables high overall acceleration factors and provides flexibility for maximizing spatial–temporal resolution and volume coverage. The high BOLD sensitivity of this hybrid MB-EVI approach and its compatibility with online image reconstruction enables high spatial–temporal resolution real-time task-based and resting state fMRI.

## Introduction

There is a need to develop fMRI data acquisition techniques at high magnetic fields to achieve both sub-millimeter spatial and sub-second temporal resolution across the whole brain, in order to unravel functional organization and connectivity at the laminar level (Petridou and Siero, 2019; Huber et al., 2021; Choi et al., 2022; Kashyap et al., 2018). Further, the organization of information flow across cortical layers has been shown to be feasible with high spatial resolution fMRI (Kashyap et al., 2018). Increases in spatial–temporal resolution are also desirable for unaliased sampling of physiological signal pulsation at high spatial resolution and for reducing sensitivity to rapid movement. While achieving millimeter spatial and sub-second temporal resolution in fMRI holds promise for advancing the characterization of Connectome dynamics, they often require compromises in spatial coverage that impair the performance of motion correction and the ability to characterize long-range connectivity across distant cortical areas. Variations in ongoing activity have been shown to predict changes in task performance and alertness, highlighting their importance for understanding the connection between brain activity and behavior (Eichele et al., 2008; Sadaghiani et al., 2010). Dynamics are potentially even more prominent in the resting-state, during which mental activity is unconstrained. As with recent studies exploring resting-state dynamics (Chang and Glover, 2010; Kiviniemi et al., 2011; Sakoglu et al., 2010), it is of interest to capture and exploit the dynamic changes and variability over time in brain activity. Monitoring these dynamics in real-time to assess data quality is expected to improve consistency of data quality in clinical research studies and the understanding of the underlying neurophysiological mechanisms. Finally, the ability to process and analyze data online and in real-time could allow for rapid optimization of acquisition paradigms and connectivity-based neurofeedback.

Recent advances in fMRI acquisition rates, using multi-band (simultaneous multislice) encoded EPI (MB-EPI) (Moeller et al., 2010; Setsompop et al., 2012), simultaneous image refocusing (Chen et al., 2015), magnetic resonance encephalography (MREG) (Hennig et al., 2020), highly accelerated echo-volumar imaging (Witzel et al., 2008, 2011) and multi-slab echo-volumar imaging (MS-EVI) (Posse et al., 2012), have played an important role in improving sensitivity for mapping functional connectomics (Feinberg et al., 2010; Smith et al., 2012; Posse et al., 2013) and for detecting task-based and resting state fMRI signal changes at frequencies above 0.2Hz (Lewis et al., 2016). In MS-EVI an entire 3D k-space with a slab is sampled following a single excitation (Mansfield et al., 1989), which accelerates the volume acquisition and must be accomplished in a time on the order of the T_2_* (~ 50 ms at 3 T) to minimize blurring and geometrical distortion. The stack of slabs to cover the volume of interest is acquired sequentially using an interleaved acquisition order to minimize signal saturation at slab edges. This approach combines the sampling efficiency of single-shot 3D encoding with the sensitivity advantage of multi-echo acquisitions (Posse et al., 2012; Puckett et al., 2018). It allows for significantly increased temporal resolution without the √*R* penalty incurred when using conventional parallel imaging methods combined with the sensitivity advantage of volumetric data acquisition. MS-EVI is synergetic with simultaneous multi-slab encoding, since it spatially resolves the phase variation at the edges of the slabs that result from simultaneous multi-slab encoding, thus increasing overall SNR and minimizing cross-talk between slabs. Further increases in spatial and temporal resolution require a combination of multiple complementary acceleration approaches. Finally, compressed sensing (CS) and deep learning-based image reconstruction has been shown to be particularly effective in combination with parallel imaging (Otazo et al., 2010; Demirel et al., 2021).

In this study, we develop a hybrid approach that combines parallel imaging accelerated MS-EVI and simultaneous multi-slab or multi-band encoding with controlled aliasing, to create multi-band accelerated MS-EVI or multi-band EVI (MB-EVI). We introduce multi-shot slab-segmented acquisitions, which shorten the duration of the EVI readout and mitigate T_2_* related degradation of slice encoding, to achieve unprecedented temporal and spatial resolution for task-based and resting-state fMRI. Exponential deconvolution of the T_2_* signal decay during the EVI readout was developed to further improve slice encoding. The objective of this study was to investigate spatial–temporal resolution limits of this approach. Secondary objectives were to perform a preliminary comparison of BOLD sensitivity with MB-EPI and assess the feasibility of mapping of resting state connectivity in different frequency bands. In addition, we studied the effect of retrospective compressed sensing (CS) reconstruction, a complementary acceleration approach, on fMRI sensitivity and investigated the compatibility of MB-EVI with NORDIC denoising (Vizioli et al., 2021; Moeller et al., 2021). Preliminary accounts of this work have been presented in abstract format (Vakamudi et al., 2018; Posse et al., 2017; Posse et al., 2022).

## Materials and methods

### Pulse sequence development

**Figure 1:**
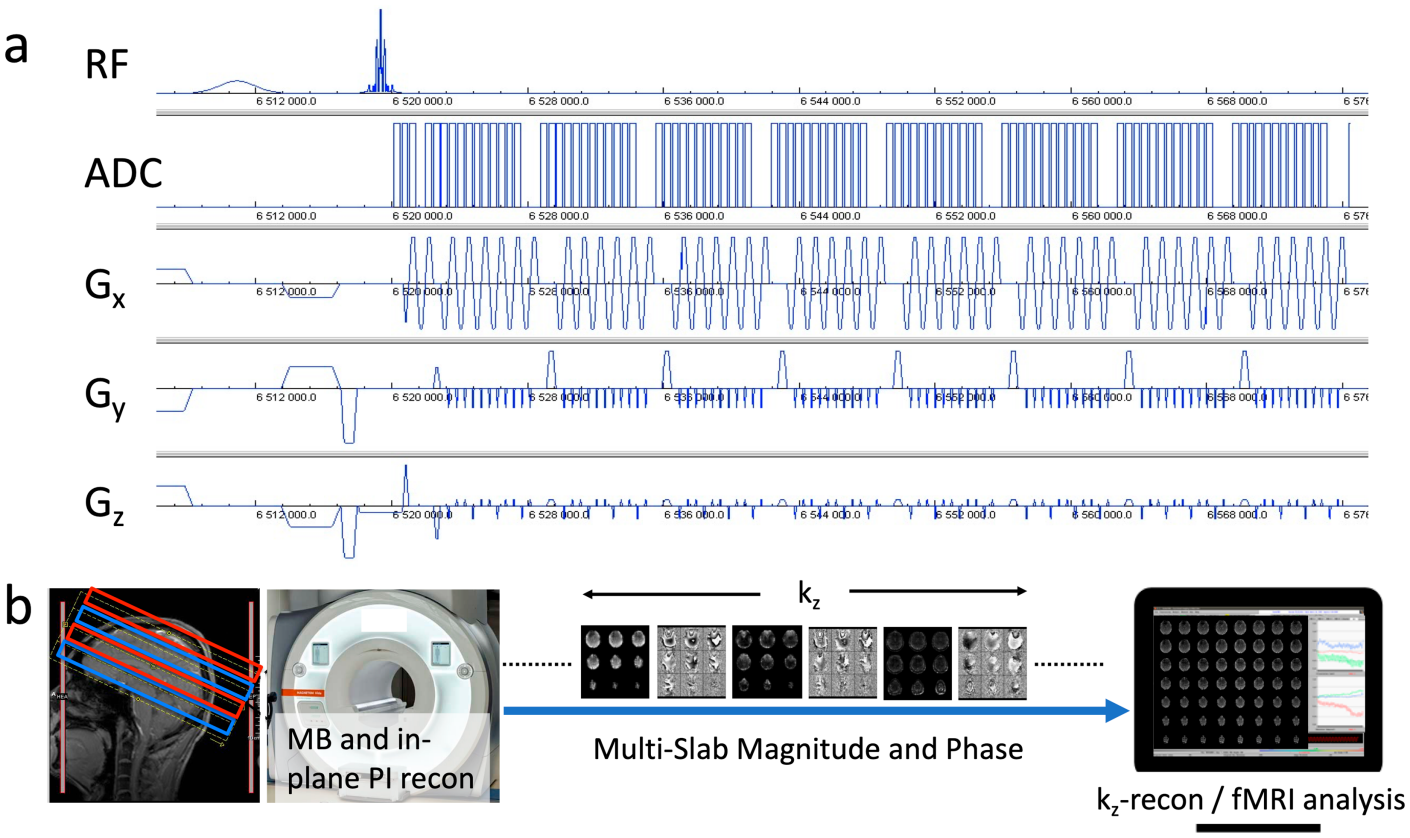
**(a)** MB-EVI pulse sequence diagram with CAIPI gradient encoding pattern. **(b)** Acquisition and reconstruction pipeline showing a multi-band acquisition of 2 sets of slabs (red/blue), multi-band (MB) and in-plane parallel imaging (PI) reconstruction on the scanner, transfer of k_z_-encoded multi-slab magnitude and phase images to an external workstation to perform k_z_-reconstruction, which consisted of the reordering of k_z_-lines from the 2 segments, a correction of T_2_* signal decay, the within-slab reconstruction of individual slices, slice reordering and slab concatenation followed by fMRI analysis.

The development of MB-EVI was performed in multiple steps on scanners with different operating systems, which allowed to implement MB-EVI with increasingly higher spatial–temporal resolution and image reconstruction performance. The initial implementation of MB-EVI on a 3 T Siemens Trio scanner with VB17A operating system used a Siemens product EPI sequence that was modified based on custom multi-echo EPI (Speck and Hennig, 1998) and MS-EVI (Posse et al., 2012) pulse sequences to support up to 32 echo times. A k_z_ dephasing gradient was implemented and k_z_ encoding gradient blips were placed in front of the EPI readout modules for each echo time to encode k_z_ steps -N/2–1 to N/2 (Figure 1a). The multi-band RF pulse from the CMRR C^2^P MB-EPI sequence (Moeller et al., 2010; Xu et al., 2013) was integrated, the maximum slice thickness was increased and a highly selective numerically optimized excitation RF pulse shape with 24 sidelobes (TSE3D_90-TB28_OP2) was implemented. RF phase increments for RF spoiling were adapted for multi-banding. Up to 4-fold in-plane GRAPPA undersampling and up to 5/8 in-plane partial Fourier (PF) acquisition were used. PF encoding of the k_z_ dimension and blipped CAIPI shifting for controlled aliasing in parallel imaging (Setsompop et al., 2012; Breuer et al., 2005) were added. The prescan consisted of a noise acquisition, measurement of auto-calibration signal lines and a single-band reference acquisition. Since the raw data format was not compatible with the CMRR C^2^P MB-EPI online reconstruction, the raw data were reconstructed offline in MATLAB using regularized “leak-block” slab-GRAPPA multiband-reconstruction, which limits the slab cross-talk between excitation shots (Todd et al., 2016), in-plane GRAPPA reconstruction and coil channel combinations.

MB-EVI was further developed on a 3 T Siemens Prisma scanner with VE11C operating system based on the widely used CMRR C^2^P MB-EPI sequence that supports multi-echo acquisition (Moeller et al., 2010; Xu et al., 2013). An important addition was a slice-segmented interleaved acquisition scheme, which was implemented by acquiring odd k_z_-lines in the first segment and even k_z_-lines in the second segment. TE-shifting between the 2 segments to achieve a more continuous T_2_* weighting of consecutive k_y_ lines was supported, which, however, increased the overall TR by twice the TE shift. All slabs of the first segment were acquired first, followed by all slabs of the second segment. Switching off k_z_ encoding for the in-plane parallel imaging reference scans (auto-calibration signal lines), while keeping k_z_ encoding for the single band and multiband scans, increased the SNR of the reference scans and reduced image ghosting. Navigator-based phase correction could be performed either jointly for both segments or separately. Several RF pulse shapes, including the TSE3D_90-TB28_OP2 pulse and the numerically optimized sinc pulses with selectable time-bandwidth product, that supported simultaneous excitation of up to 6 slabs were available. Additional selectable options included: Multi-band RF phase scrambling, dual slice GRAPPA kernels (Setsompop et al., 2012), SNR optimized (SENSE1) magnitude coil combination instead of sum of squares (Sotiropoulos et al., 2013), 6/8 slice PF acquisition, blipped CAIPI shifting (on by default), phase correction scans for both even and odd k_z_ segments (instead of using the phase correction scans of the first segment for the second segment). Another important addition was the development of an online reconstruction pipeline that was distributed between the scanner reconstruction computer and an external workstation. The multi-band and in-plane reconstruction was performed on the scanner using the CMRR C^2^P MB-EPI reconstruction pipeline with regularized “leak-block” slab-GRAPPA multiband-reconstruction and unaliasing of blipped CAIPI shifting (Figure 1b). A custom real-time image export functor was inserted at the end of the scanner reconstruction pipeline to transfer a partial image header file and a series of partly reconstructed 4D data (x, y, k_z_, slab) in form of mosaic raw magnitude and phase images via TCP/IP to an external workstation in real-time for online EVI reconstruction and fMRI analysis using the custom TurboFIRE real-time fMRI reconstruction and analysis software (Posse et al., 2012; Posse et al., 2013; Posse et al., 2001). The online image reconstruction steps on the external workstation included reordering of k_z_-lines from the 2 segments, the within-slab reconstruction of individual slices, slice reordering, slab concatenation and combination of the inner slices from each of two adjacent slabs in case they overlapped in the gaps. In addition, a correction for T_2_* signal decay during the EVI readout that degrades k_z_ encoding was implemented in TurboFIRE. The k_z_ data within a segment were amplitude corrected assuming a spatially invariant T_2_* decay. The correction factor A in Equation (1) was applied to the raw magnitude images:


(1) $$A\;\left(k_{z}\right)=\exp\left(TE\left(k_{z}\right)/{T_{2}}^{*}\right)$$

where a user selectable average T_2_* value for gray matter of 66 ms (Peters et al., 2007) was employed. This correction increased the output image dynamic range from 16 to 18 bit.

Online processing at the UNM site was performed on an Intel Xeon E5-2697 v4 @ 2.30 GHz × 51, 2 × 18 core workstation with 64 GB RAM and 64Bit Ubuntu operating system 16.04. Online processing at the UMN site was performed on a 2.8 GHz quad-core MacBook Pro laptop with 16 GB RAM and 64Bit Ubuntu 16.04 running in a VMWare virtual machine.

While a wide range of parameter combinations for multi-band and in-plane parallel imaging acceleration, spatial and temporal resolution, and volume coverage were supported, specific parameter combinations that pushed the limits of spatial resolution, temporal resolution and both on the two scanner configurations were used (Table 1). Without slab-segmented acquisition, a maximum nominal in-plane resolution of 1.5 mm was feasible, while slab-segmented acquisition enabled a maximum nominal in-plane resolution of 1 mm. For comparison, experiments were performed with multi-band factor: 1, which corresponds to MS-EVI (Posse et al., 2012), and with MB-EPI to assess image quality and fMRI sensitivity of MB-EVI. G-factor related noise enhancement (Breuer et al., 2009) was characterized for selected acceleration factors.

**Table 1:** Selected MB-EVI and MB-EPI pulse sequence parameter combinations.

Pulse sequence	MB-factor	No. slabs	GRAPPA factor (k_y_)	Slab segmentation	Partial k_y_	Partial k_z_	TR [ms]	TE_eff_ [ms]	Flip angle [degrees]	Voxel size - nominal [mm^3^]	In-plane image matrix	Number of slices (reconstructed)*
MB-EVI	1	6	4	No	6/8	No	436	29	50	3 × 3 × 3	64 × 64	48
MB-EVI	3	6	4	No	6/8	No	163	29	10	3 × 3 × 3	64 × 64	48
MB-EVI	1	9	4	No	6/8	No	904	29	17	3 × 3 × 3	64 × 64	64
MB-EVI	3	9	4	No	6/8	No	302	29	12 or 37	3 × 3 × 3	64 × 64	64
MB-EPI	8	N/A	0	N/A	6/8	N/A	745	29	14	3 × 3 × 3	64 × 64	72
MB-EVI	5	10	4	No	6/8	No	145	29.5	10	3.1 × 3.1 × 3	64 × 64	71
MB-EVI	1	12	4	No	6/8	No	1140	40.5	70	2.5 × 2.5 × 2	80 × 80	85
MB-EVI	3	12	4	No	6/8	No	388	40.5	40	2.5 × 2.5 × 2	80 × 80	85
MB-EVI	3	9	4	No	6/8	6/8	302	30	12	2 × 2 × 2	98 × 98	64
MB-EPI	8	N/A	0	N/A	6/8	N/A	745	29	14	2 × 2 × 2	98 × 98	72
MB-EVI	3	9	4	No	6/8	6/8	334	29	10	1.5 × 1.5 × 1.5	128 × 128	64
MB-EVI	4	8	4	Yes	6/8	6/8	530	37	25	1 × 1 × 1	192 × 192	57
MB-EVI	4	8	4	Yes	5/8	6/8	508	31	25	1 × 1 × 1	192 × 192	57
MB-EPI	2	N/A	3	N/A	5/8	N/A	932	30	55	1 × 1 × 1	192 × 192	24**

* After slab concatenation. ** Inter-slice gap = slice thickness.

### Experiments

#### Technical improvements

Pulse sequence optimization was performed on spherical quality assurance phantoms and *in vivo*. MB-EVI was acquired with MB factors ranging from 1 to 4, isotropic nominal voxel sizes ranging from 1 to 6 mm, number of slabs between 4 and 12, number of slices per slab: 8 and flip angles ranging from 10° to the Ernst angle for gray matter (Table 1). A temporal resolution of 145 ms with 3 mm spatial resolution and whole brain coverage was feasible using multi-band-factor 5. An isotropic resolution of 1.5 mm with 64 reconstructed slices was encoded without k_z_-segmentation, 6 k_z_-steps (6/8 partial k_z_ encoding) and an EVI readout duration of 86.64 ms, enabling a minimum TR of 334 ms. An isotropic resolution of 1 mm with 127 reconstructed slices was encoded using k_z_-segmentation and multi-band factor: 3, 3 k_z_-steps per segment and an EVI readout duration of 90 ms, enabling a minimum TR of 1720 ms. Shorter TRs were achieved using a larger multi-band factor of 4 and smaller volume coverage: TR/TE_eff_: 650/40 ms (GRAPPA factor: 3) and 500/31 ms (GRAPPA factor: 4), voxel size 1 mm isotropic, number of reconstructed slices: 57. The readout bandwidth ranged from 2,790 Hz/pixel for a 3 mm voxel size to 1,302 Hz/pixel for a 1 mm voxel size.

A retrospective simulation of further acceleration with compressed sensing was performed offline using fully reconstructed MB-EVI data (multiband factor: 3) that were inverse Fourier transformed. Compressed sensing was implemented using variable-density random undersampling based on a Poisson disk distribution on k_y_-k_z_ of the full slab k-space data and nonlinearly reconstructed using the SPARSE-SENSE algorithm (Otazo et al., 2010) with temporal PCA (Chen and Feinberg, 2013) as the sparsifying transform. The CS reconstruction parameter was chosen to minimize spatial–temporal smoothing by visual inspection. All processing was performed on a Linux workstation equipped with an Intel(R) Xeon(R) CPU E5530 and 24GB memory.

NORDIC denoising (Vizioli et al., 2021; Moeller et al., 2021) was integrated into the offline MATLAB raw data reconstruction pipeline described above to assess spatial smoothing and increase in fMRI sensitivity and applied to two selected MB-EVI data sets with 4 mm and 2 mm isotropic resolution. NORDIC was applied to remove components that could not be distinguished from gaussian noise. A locally low-rank SVD with hard Singular value thresholding based on zero mean i.i.d. noise (Moeller et al., 2021) was applied on patches with an 11:1 ratio between spatial and temporal voxels, and a stride of 1/2 the patch size between overlapping patches. The data was reconstructed with in-house written MATLAB code. NORDIC was applied using code available via.^1^ All processing was performed on a linux workstation equipped with an Intel(R) Xeon(R) CPU E5 and 128GB memory.

#### Applications

Task-based (motor/visual) and resting-state data were acquired in 21 healthy controls and a male patient with low grade glioma on a 3 T Siemens Trio scanner using a 32-channel head coil and on 3 T Siemens Prisma scanners equipped with a 32-channel head array coil. Informed consent was obtained using human subject protocols approved by the IRBs at the University of New Mexico and the University of Minnesota. For comparison, data with identical nominal voxel size was acquired using the CMRR C^2^P MB-EPI sequence with dual-echo acquisition (TR/TE1/TE2: 400/14/41 ms, flip angle: 40°, multi-band factor: 8, GRAPPA factor: 1, voxel size: 3 mm isotropic, 32 slices) and single-echo acquisition (TR/TE:932/30 ms, flip angle: 55^o^ multi-band factor: 2, GRAPPA factor: 3, voxel size: 1 mm isotropic, no slices: 24, slice gap: 100%, readout bandwidth: 1240 Hz/pixel). Data were acquired using the Siemens advanced shimming mode.

### fMRI data analysis

Task- and resting-state fMRI analysis using the TurboFIRE fMRI software tool (Posse et al., 2013; Posse et al., 2001) included the following preprocessing steps: six-parameter rigid body motion correction, isotropic spatial smoothing using a Gaussian kernel (ranging from 0 to 2 mm smoothing for 1 mm isotropic voxel size to 5 mm smoothing for 3 and 4 mm nominal voxel size), spatial normalization using manual selection of the midpoint of the AC/PC line and mapping of the Montreal Neurological Institute (MNI) atlas into subject space using a lookup table approach (Gao and Posse, 2003), and an 8 s moving average time domain low pass filter with a 100% Hamming window width (Lin et al., 2012) to reduce signal fluctuations due to cardiac and respiratory pulsations. The lookup table related coordinates in object space to MNI space used voxel to voxel mapping. Since several voxels in normalized space may be projected to the same voxel in object space, corresponding source locations in normalized space were spatially averaged. The initial 10 s of data were discarded to account for non-steady-state signal changes. A maximum of 12 regions of interest (ROI) were simultaneously processed in TurboFIRE to generate time courses for correlation analysis (Gao and Posse, 2004). Brodmann areas (BAs) were selected as seed regions after transforming the coordinates from the MNI atlas into the Talairach atlas using Matthew Brett’s formula^2^ and automatically assigned using a modified Talairach Daemon database (Lancaster et al., 2000). For real-time analysis, a 10 s prescan was spatially normalized to select atlas-based seed regions. WM and CSF seeds for regression were manually delineated. Resting-state networks (RSNs) were mapped using unilateral Brodmann area (BA) based seed regions [auditory- AUN–BA4142; default-mode-DMN–BA731; sensorimotor-SMN–BA01-03, visual-VSN–BA17, language-LAN-BA4445 (Broca) and BA223940 (Wernicke)].

Additional analyses were carried out using spatial ICA with the GIFT toolbox^3^ for the purpose of denoising to remove respiratory pulsation, to detect task activation and to map resting-state connectivity.

## Results

### Technical improvements

**Figure 2:**
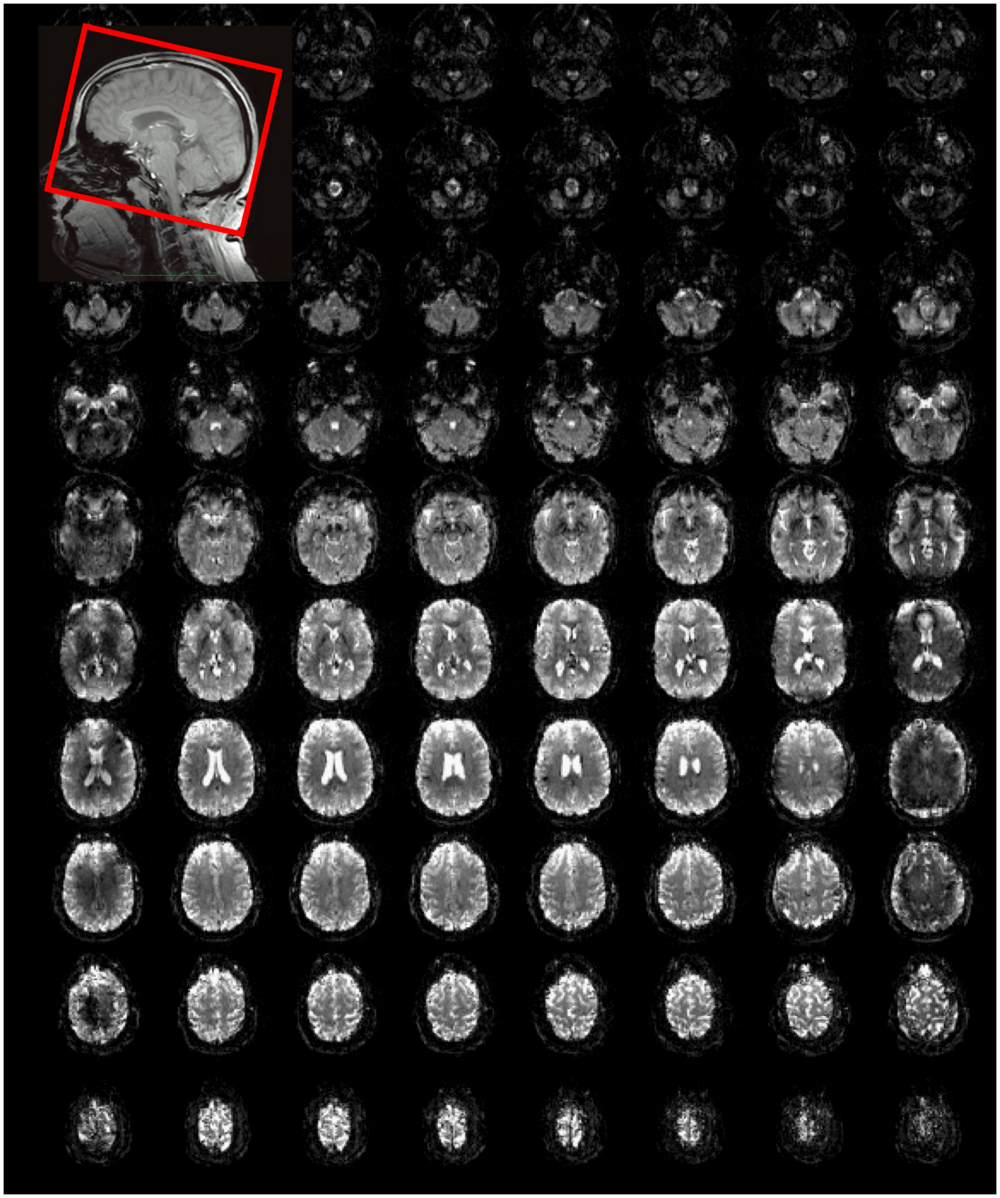
Whole brain MS-EVI (multi-band factor: 1) with 2.5 × 2.5 × 2 mm^3^ spatial resolution. TR/TE: 1,140/40.5 ms, flip angle: 70°, 12 slabs, slab thickness: 14.5 mm, slab gap: 1.5 mm. A subset of 10 slabs and all 8 slices of each slab are displayed. Slab concatenation was not applied, since there was no overlap between neighboring slices in adjacent slabs. The inset at the top left shows the localization of the slab stack. The yellow arrows depict the non-uniform image intensity at the edges of the slabs.

**Figure 3:**
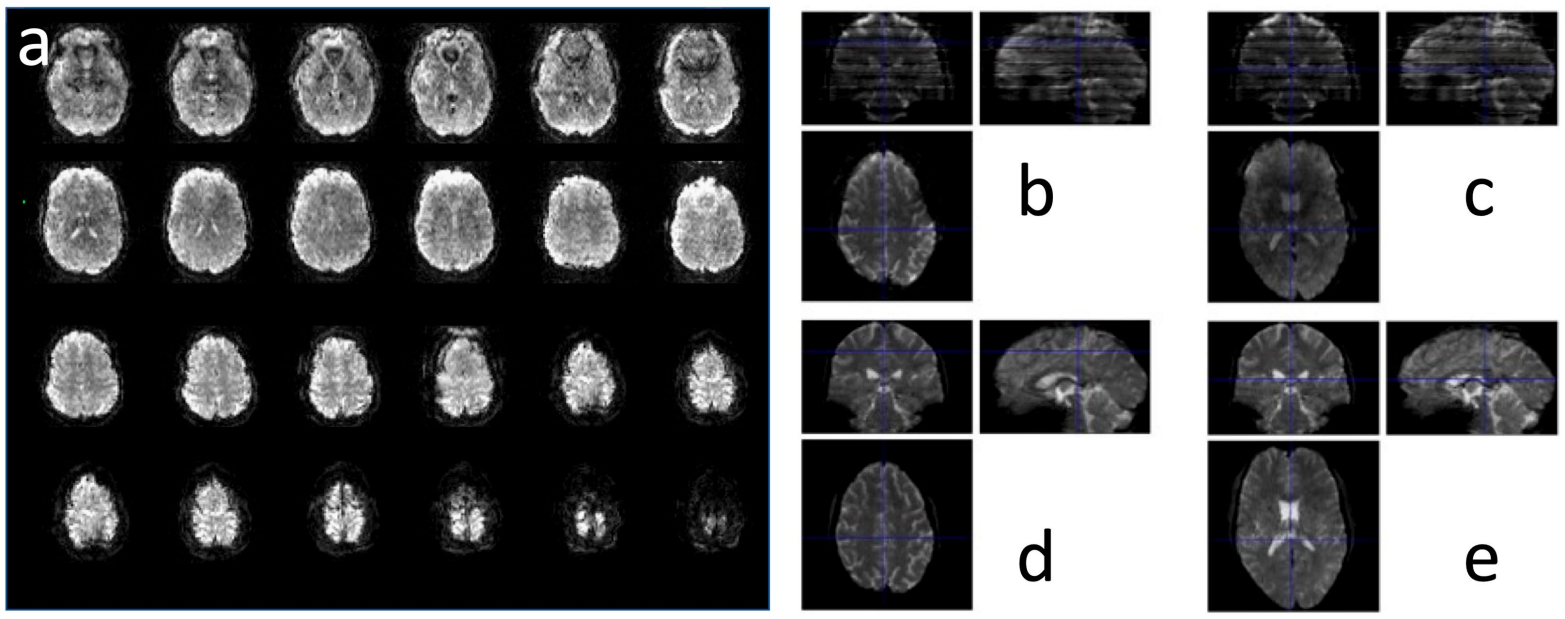
MB-EVI in a healthy control at 2 mm isotropic resolution showing **(a)** all 8 slices of the 9 slabs and **(b,c)** a comparison with **(d,e)** MB-EPI in 2 slice locations. MB-EVI: TR/TE: 302/30 ms, flip angle: 12°, multi-band factor: 3, 9 slabs, 64 reconstructed slices. MB-EPI: TR/TE: 745/29 ms, flip angle: 14°, multi-band factor: 8, 72 slices.

**Figure 4:**
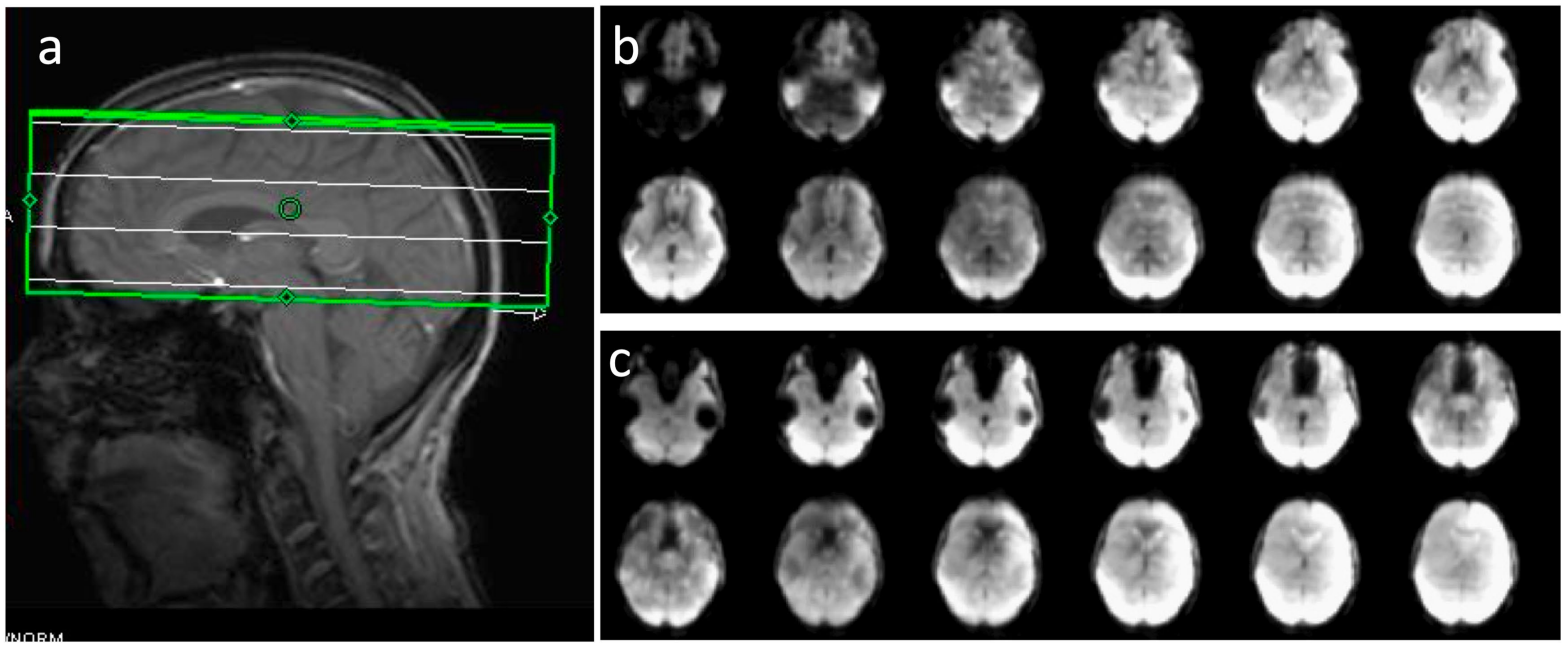
Sensitivity of MB-EVI to magnetic field inhomogeneity as a function of k_z_-encoding trajectory. **(a)** Volume prescription for a 4-slab acquisition with 3 × 3 × 3 mm^3^ voxel size. **(b)** Slices in the lower 2 slabs with inversion of k_z_ encoding gradients, which decreases signal in lateral orbital-frontal and in parts of occipital lobe, but enhances signal in medial orbital-frontal cortex and in temporal lobe. **(c)** Reversal of the k_z_ encoding gradients restores signal in lateral orbital-frontal and occipital lobe visual cortex, but descreases signal in medial orbital-frontal cortex and in temporal lobe.

**Figure 5:**
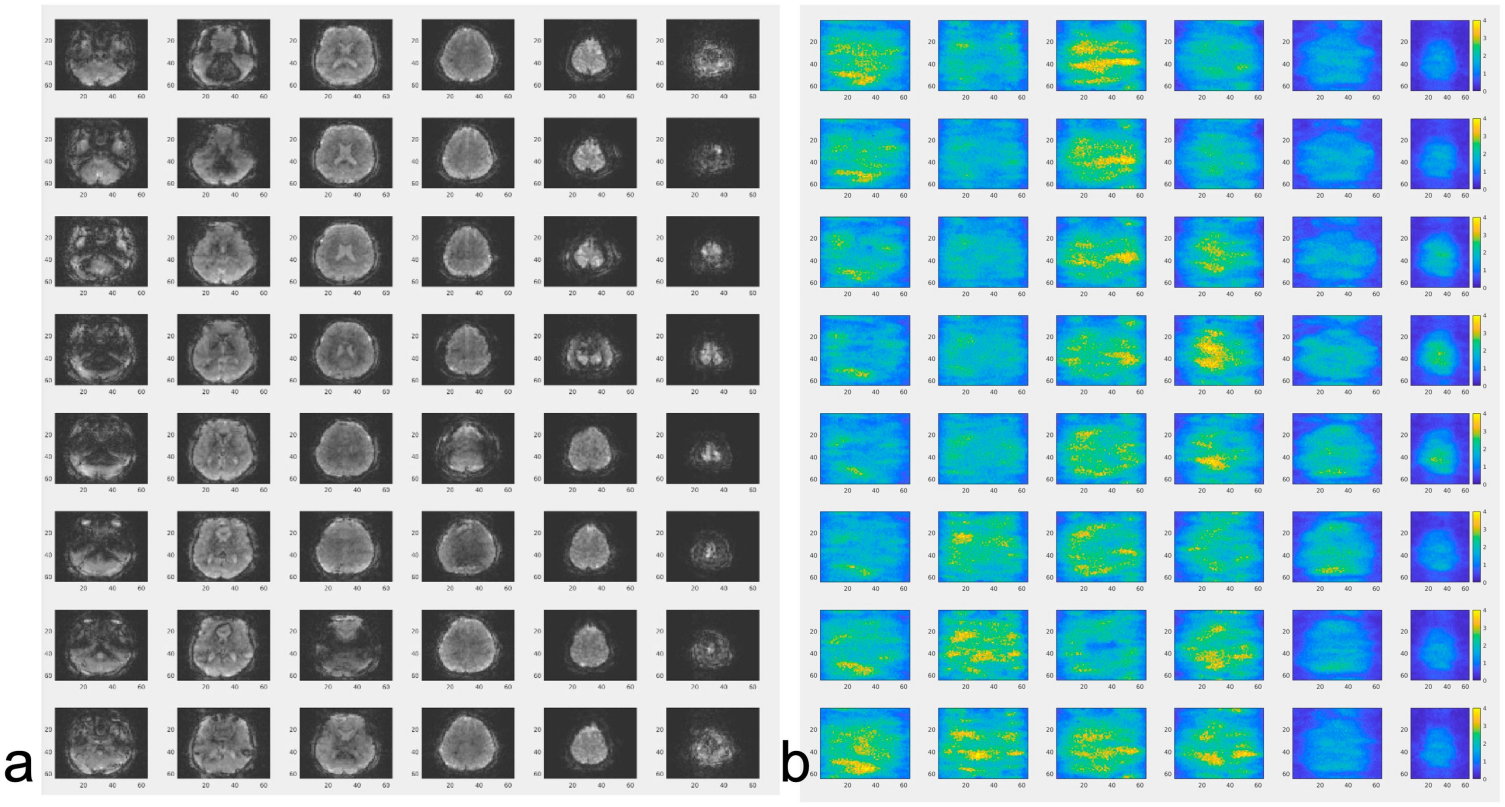
**(a)** MB-EVI and **(b)** corresponding g-factor maps (scaled 0–4) *in-vivo*. TR/TE: 154/33 ms, flip angle: 10^o^, multi-band factor: 3, 6 slabs (displayed vertically), 3 mm isotropic spatial resolution.

The image quality obtained using MS-EVI (i.e., MB-EVI with multi-band factor 1) with full k_z_-encoding and 4-fold GRAPPA acceleration served as a benchmark for MB-EVI sequences. An example of whole brain coverage with 2.5 × 2.5 × 2 mm^3^ voxel size is shown in Figure 2. Uniform image intensity within slabs except for the edge slices was obtained. Signal loss in orbital frontal cortex and temporal lobes was consistent with the TE of 40.5 ms and not much stronger than in MB-EPI. The addition of multi-band acceleration and PF encoding in k_z_ increased ghosting in the first phase encoding direction and image non-uniformity. Ghosting increased strongly beyond 4-fold multi-band acceleration. The signal losses and intensity non-uniformity at the edges of the slabs increased with decreasing TR, necessitating negative inter-slab gaps and combination of slices from adjacent slabs to improve volume coverage. These signal losses and intensity non-uniformity were mitigated in part using highly selective RF pulses with narrow transition bands at the slab edges. The in-plane image resolution and in-plane geometrical distortion of MB-EVI was comparable to that of MB-EPI at the same voxel size (Figure 3). T_1_ signal saturation, which was stronger in MB-EVI than in MB-EPI due to the shorter TR, attenuated signals in cerebrospinal fluid filled spaces. The signal loss at the edges of the slab profiles modulated the image intensity in the sagittal plane, creating a striping pattern in the reconstructed volume images when viewed in the sagittal and coronal planes. Increasing spatial resolution significantly decreased the sensitivity to B_0_ inhomogeneity. Geometrical distortion in the slice encoding direction due to B_0_-inhomogeneity was seen in the most superior slabs, in the orbital-frontal cortex and in the cerebellum (Figure 3). At lower spatial resolution, the signal losses in the orbital-frontal cortex and in temporal lobe increased. These could be recovered by inverting the k_z_-encoding gradients at the expense of signal losses in other brain regions (Figure 4). CAIPI shifting and reconstruction with the initial version of MB-EVI on the Trio scanner substantially increased ghosting in the in-plane phase encoding direction. All subsequent multi-band accelerated results on the Trio scanner were thus acquired without CAIPI shifting. As a consequence, the g-factors for this version were higher than those of typical multi-band EPI sequences. For a multi-band factor of 2 the mean g-factor was 2.5 ± 1.3. For a multi-band factor of 3 the g-factor ranged from 1.5 in the top slab to 4 in central slabs with a mean of 2.8 ± 0.6 (Figure 5). A comparison of the offline MATLAB recon and the online recon of single-band multi-slice EVI data showed that the offline MATLAB recon had slightly stronger image intensity nonuniformity and ghosting from double images that were shifted by ½ FOV along the in-plane phase encoding direction.

**Figure 6:**
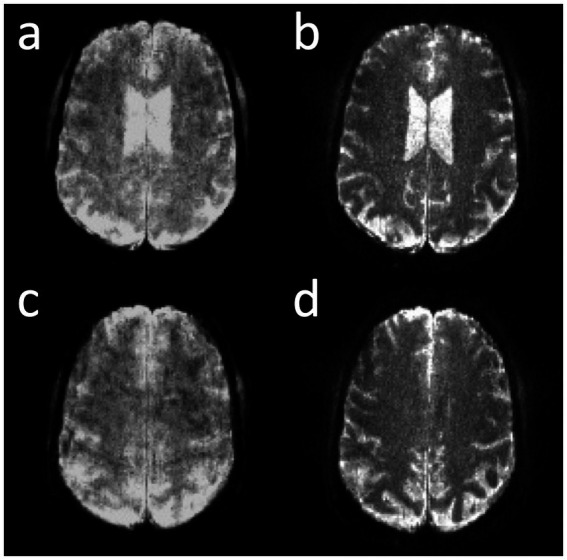
Online correction of T_2_* signal decay in MB-EVI with 1 mm isotropic nominal voxel size in two slice locations. **(a,c)** Before and **(b,d)** after correction.

Correction of the T_2_* signal decay during the EVI readout was investigated to show proof-of-concept of significantly increased spatial resolution and contrast in the reconstructed images, which, however, as expected decreased the SNR (Figure 6).

**Figure 7:**
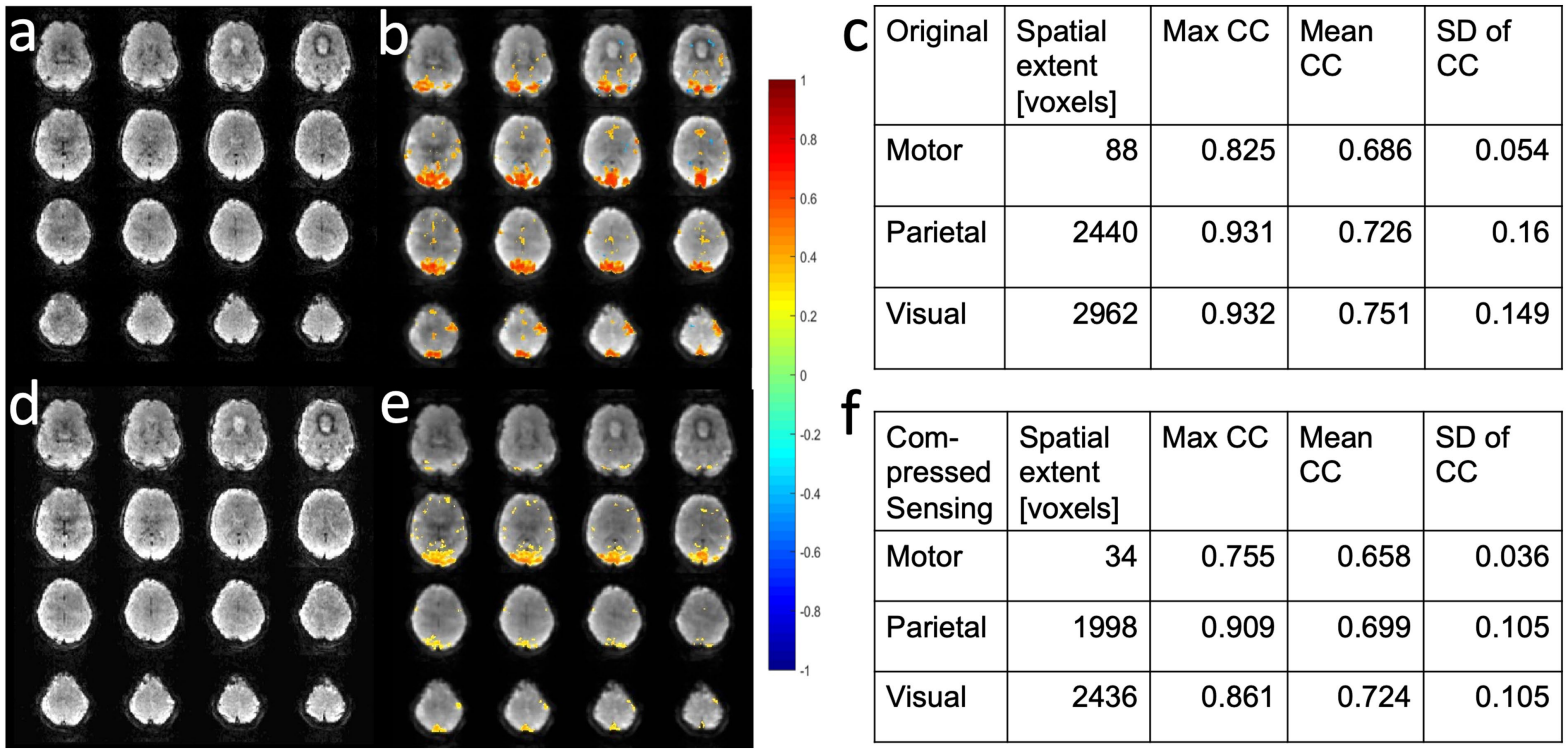
Retrospective compressed sensing of task-based fMRI acquired using MB-EVI. **(a–c)** Original fully reconstructed MB-EVI data and **(d–f)** retrospective compressed sensing with 2.4-fold acceleration of MB-EV data. **(a,d)** Raw images. **(b,e)** Seed-based correlation map of visual activation. **(c,f)** Cluster analysis of task-based correlation in motor, parietal and visual cortices. TR/TE_eff_: 163/35 ms, flip angle: 28^o^, multi-band factor: 3, voxel size: 3 mm isotropic, number of slabs: 6, reconstructed number of slices: 43, number of scans: 1070 (3 min scan). Correlation coefficient (CC) threshold: 0.5.

**Figure 8:**
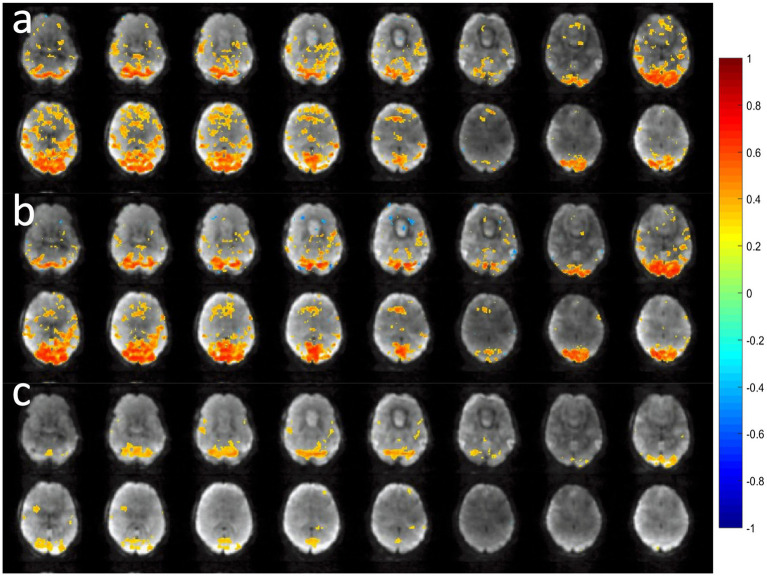
Retrospective compressed sensing reconstruction using 2-fold acceleration with different regularization factors *λ* of a MB-EVI scan acquired during task activation: **(a)** λ = 1, **(b)** λ = 5, and **(c)** λ = 9. Activation maps are overlaid on raw MB-EVI images. TR/TE_eff_: 163/35 ms, flip angle: 28^o^, multi-band factor: 3, voxel size: 3 mm isotropic, number of slabs: 6, reconstructed number of slices: 43, number of scans: 1,070 (3 min scan). Correlation threshold: 0.5.

Compressed sensing with 2.4-fold retrospective undersampling relative to the fully sampled k-space showed negligible loss in image quality and moderate region-specific losses in BOLD sensitivity of task activation (Figure 7). Similar image quality but increased spatial blurring of activation patterns was measured using 3.1-fold undersampling. Sensitivity to task activation using 2-fold undersampling increased with regularization up to a regularization factor *λ* of 5, beyond which spatial blurring of the activation pattern increased and sensitivity decreased (Figure 8).

**Figure 9:**
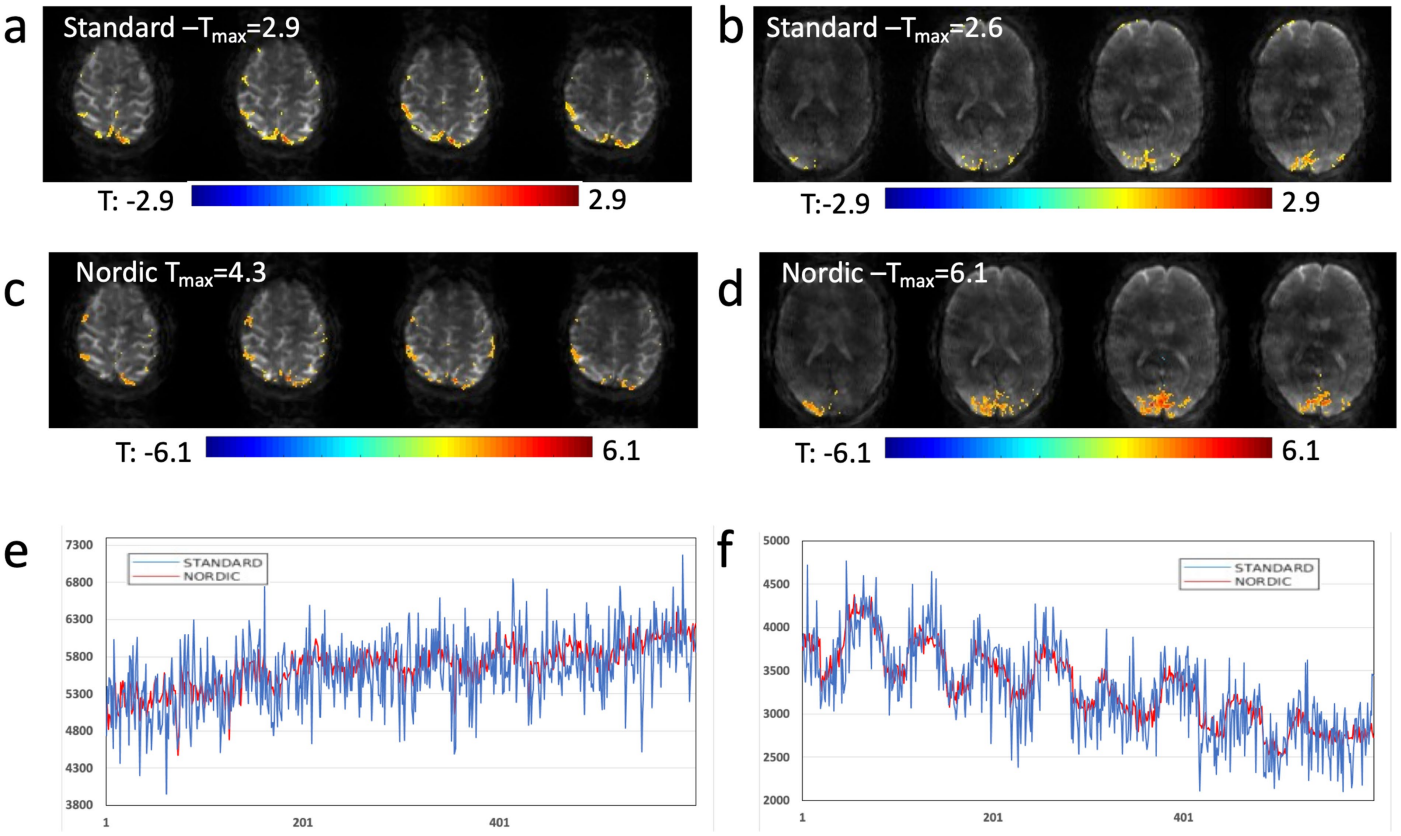
NORDIC denoising of high spatial and temporal resolution MB-EVI during motor/visual task. **(a)** Motor and **(b)** visual activation using standard reconstruction and T-threshold of 1.25. **(c)** Motor and **(d)** visual activation using reconstruction with NORDIC denoising and t-threshold of 3.0. **(e)** Single voxel time course in frontal cortex using standard processing (blue) and NORDIC denoising (red). **(f)** Single voxel time course in occipital cortex using standard processing (blue) and NORDIC denoising (red). TR/TE: 302/26 ms, isotropic voxel size: 2 mm, flip angle: 10^o^, Number of scans: 600 (3:15 min scan time). Neither spatial nor temporal filters were used in postprocessing.

NORDIC denoising of MB-EVI scans measured with 302 ms temporal resolution and 2 mm isotropic nominal voxel size increased the temporal SNR in supraventricular white matter almost 4-fold, from 24 to 84 on average, without changing image resolution and contrast (Figure 9). The maximum T-scores increased significantly with NORDIC, approximately 1.5-fold in the motor cortex and almost 2.4-fold in visual cortex. Resting-state networks measured with 136 ms temporal resolution and 4 mm isotropic nominal voxel size were mapped with increased spatial extent and maximum correlation coefficients of major network nodes after NORDIC denoising, but with smaller gains compared to the 2 mm isotropic nominal voxel size data.

### Applications

**Figure 10:**
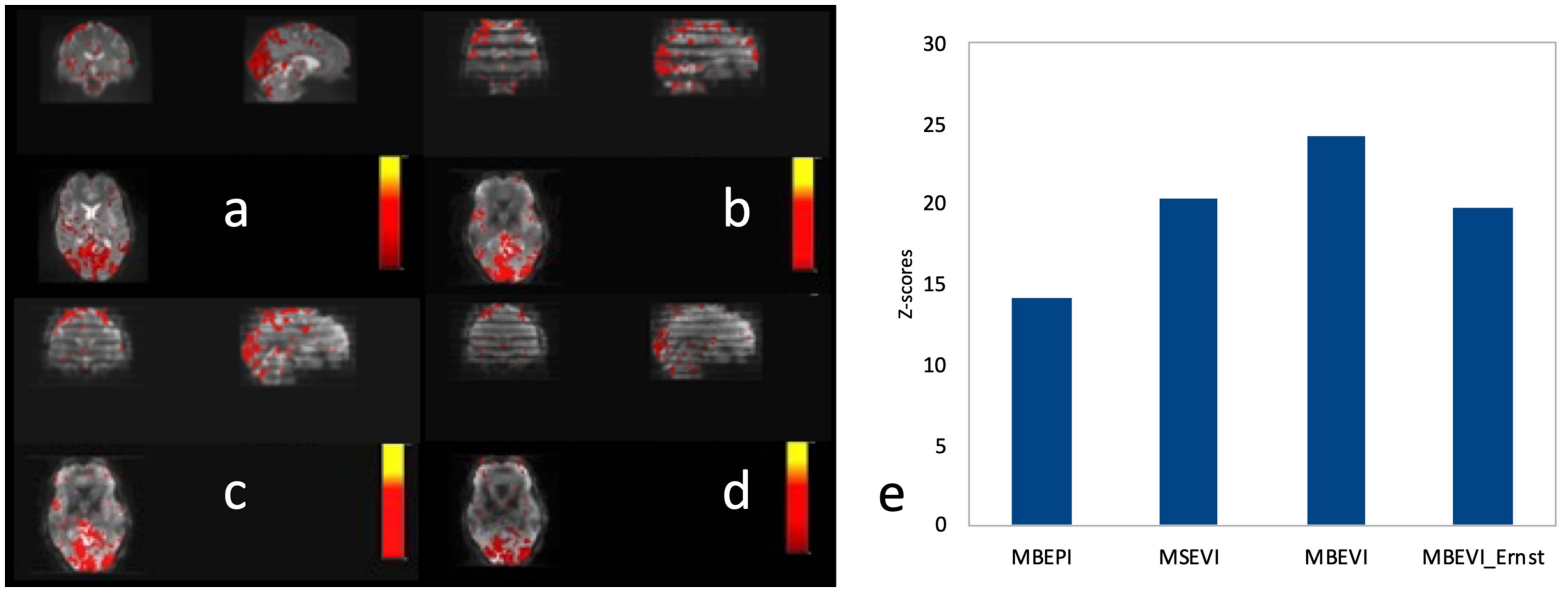
Comparison of task-based fMRI in visual cortex at 2 mm isotropic resolution using **(a)** MB-EPI, **(b)** MS-EVI **(c)** MB-EVI with flip angle: 12° and **(d)** MB-EVI using Ernst angle for gray matter: 37°. **(e)** Maximum Z-scores of visual activation. MB-EPI: TR/TE: 745/29 ms, multi-band factor: 8, flip angle: 14°, 72 slices. MS-EVI: TR/TE: 904/29 ms, flip angle: 17°, multi-band factor: 1, 9 slabs, 64 reconstructed slices. MB-EVI: TR/TE: 302/29 ms, flip angle: 12° or 37°, multi-band factor: 3, 9 slabs, 64 reconstructed slices. Visual attention task (eyes open/closed) during 3:15 min scans.

**Figure 11:**
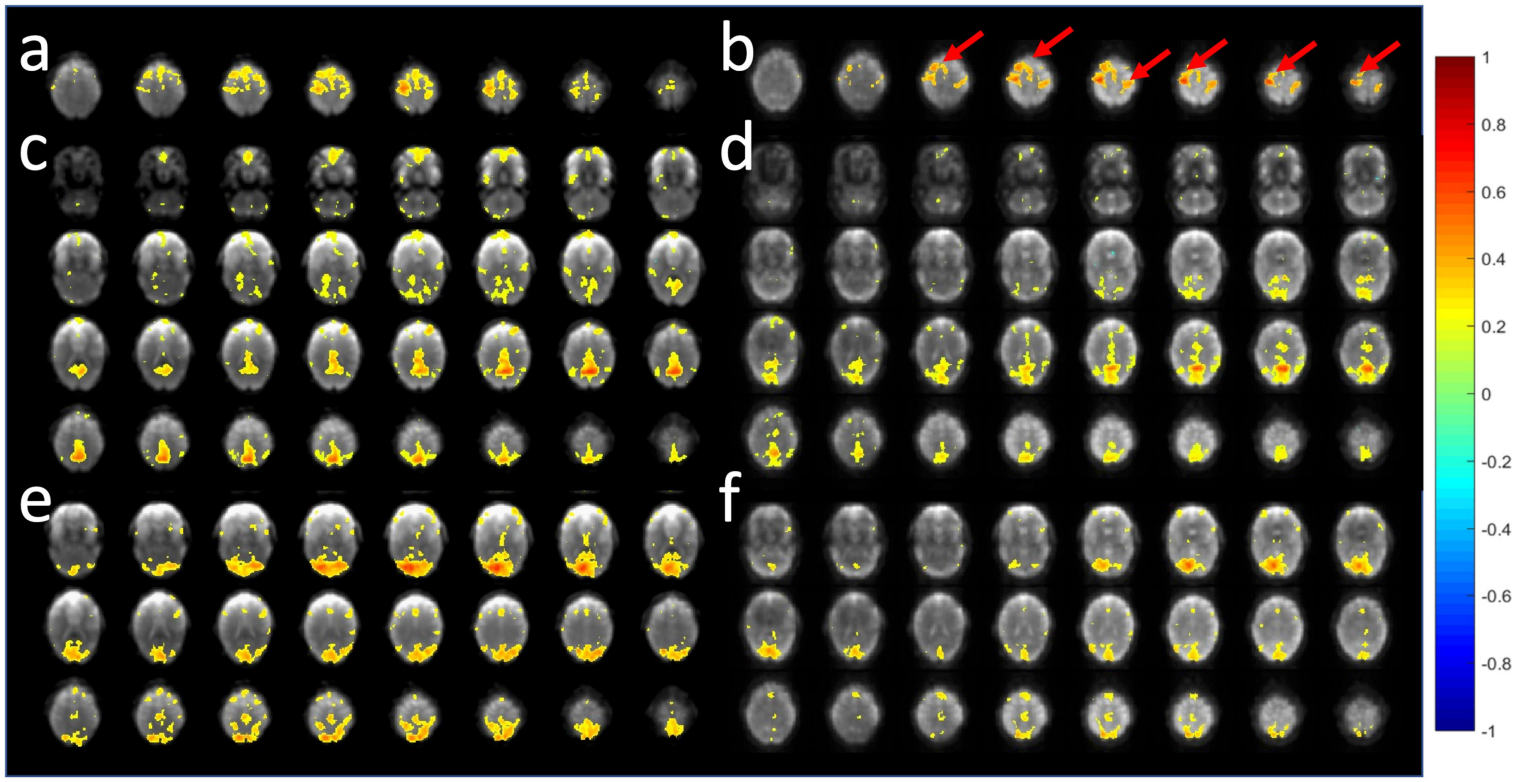
Comparison of resting-state fMRI using 6-slab EVI **(a,c,e)** without and **(b,d,f)** with 3-fold multi-band acceleration. MS-EVI: TR/flip angle/multi-band factor: 436 ms/50°/1. MB-EVI: TR/flip angle/multi-band factor: 163 ms/10°/3. TE: 30.5 ms, slab thickness: 21.6 mm, slab gap: 2.3 mm. SNR in visual cortex in these scans was similar: MS-EVI: 101 and MB-EVI: 92. Slab concatenation was not applied, since there was no overlap between neighboring slices in adjacent slabs. The red arrows depict increases in resting-state connectivity.

**Figure 12:**
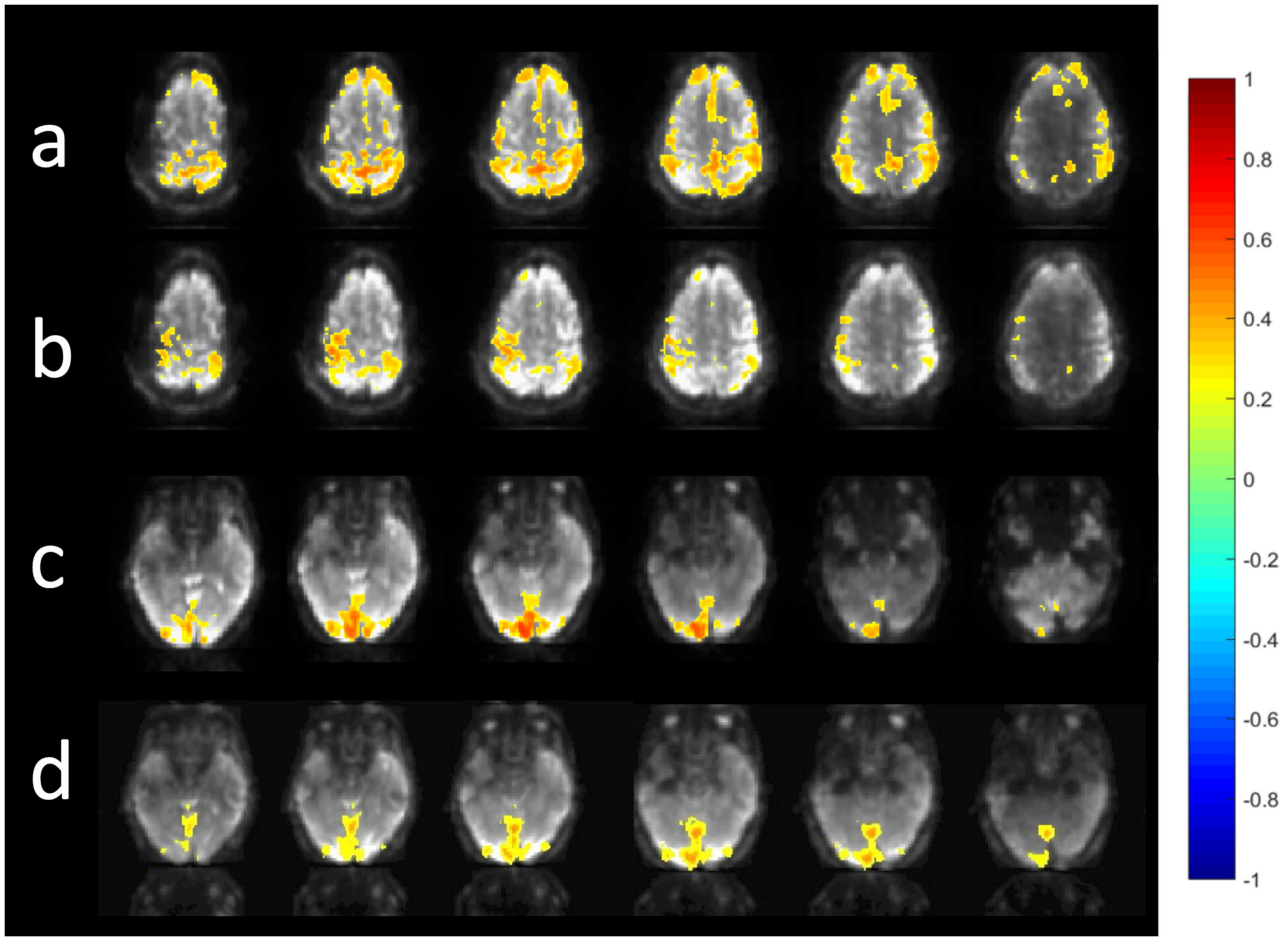
Resting state connectivity in different frequency bands using MB-EVI. Low frequency (<0.3 Hz) resting state networks: **(a)** Default mode **(b)** Sensorimotor **(c)** Visual. (Correlation threshold: 0.4). **(d)** High frequency resting state connectivity in visual cortex (filter passband 0.35–0.95 Hz, filter order: 175, correlation threshold: 0.25). Acquisition parameters for MB-EVI: TR/TE_eff_: 163/35 ms, flip angle: 10^o^, multi-band factor: 3, 6 slabs, 48 slices, voxel size: 3 × 3 × 3 mm^3^, 6 min scan.

**Figure 13:**
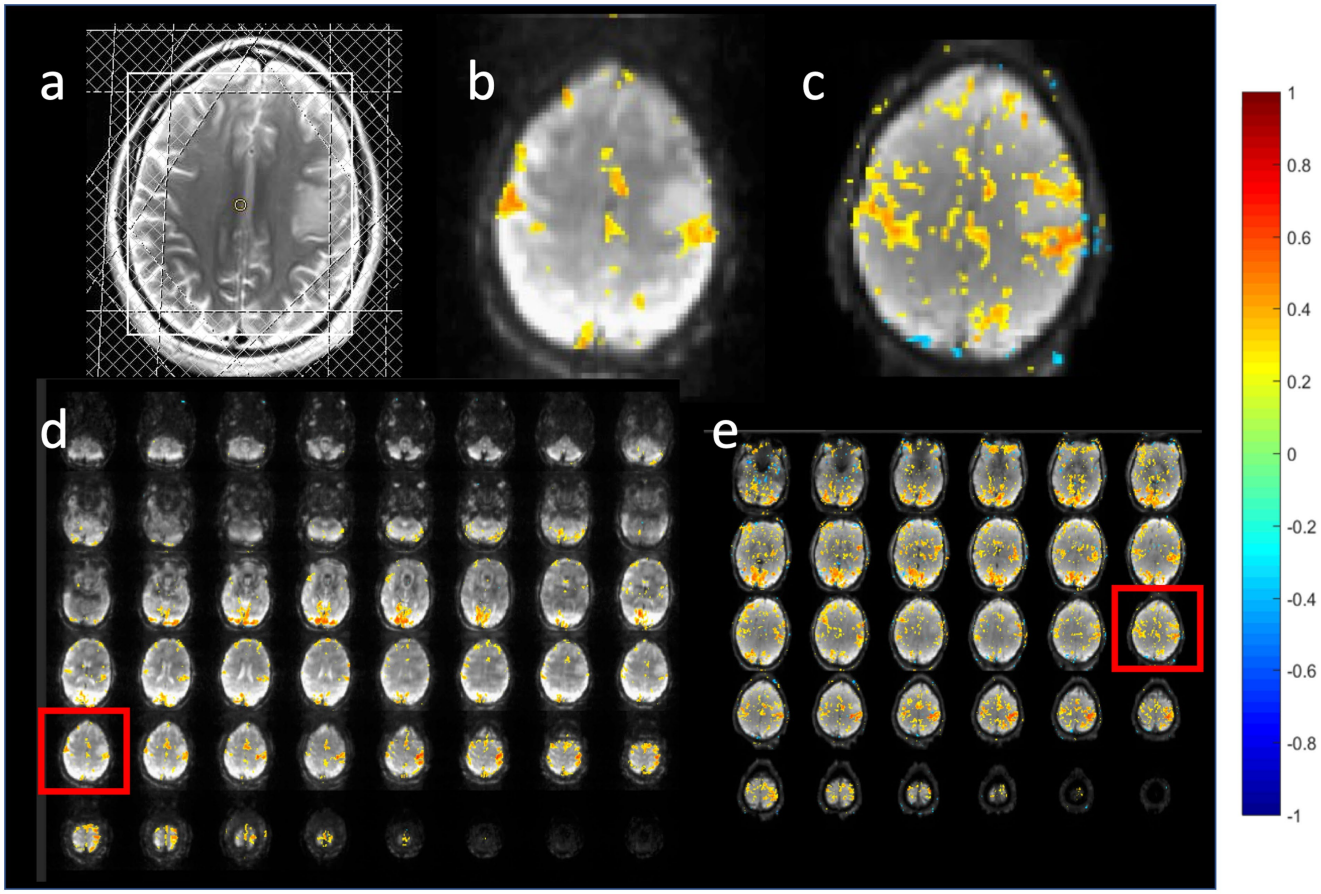
Task-based activation in a patient with a low grade glioma comparing **(b,d)** MB-EVI with **(c,e)** double-echo MB-EPI during simultaneous bilateral finger tapping and eyes open versus closed block-design paradigm. **(a)** T2-weighted MRI. The slice location of the images in **(b,c)** are shown as red boxes in **(d,e)**. Common parameters: TR: 400 ms, voxel size: (3 mm)^3^, number of images: 450 (3 min scan). MB-EVI: TE: 33 ms, flip angle: 36^o^, multi-band factor: 2, 57 reconstructed slices. Double-echo MB-EPI: TE1/TE2: 14/41 ms, flip angle: 40°, multi-band factor: 8, 32 slices. Task-based fMRI analysis was performed using weighted echo averaging. Correlation-threshold: 0.4.

A comparison of the sensitivity of MB-EPI, MS-EVI and MB-EPI for task-based activation and resting-state connectivity was performed using (i) matched volume coverage at minimum TR of each of the sequences, (ii) matched spatial SNR and (ii) matched TR. Both single-echo and dual-echo MB-EPI sequences were used. When comparing task-based activation in visual cortex measured with MB-EPI, MS-EVI and MB-EVI acquired with similar flip angles, the maximum Z-score of MB-EVI was larger than that of MS-EVI, which was larger than that of MB-EPI (Figures 10a–c,e). The spatial extents of activation measured with these sequences were similar. However, increasing the flip angle of MB-EVI to the Ernst angle (Figures 10d,e) decreased sensitivity and increased image artifacts at the slab edges. Low flip angles were thus used for all subsequent MB-EVI scans (Table 1). Comparing MS-EVI and MB-EVI with multi-band-factor 3 that were matched for spatial SNR showed that MB-EVI had slightly larger intensity nonuniformity at the edges of the slabs. Resting-state connectivity with MS-EVI was stronger than with SNR-matched MB-EVI except for the sensorimotor network (Figure 11). Sensitive mapping of major resting-state networks (RSNs) with 3 mm^3^ voxel size was feasible in multiple frequency bands up to 0.95 Hz (Figure 12). Comparing 4-fold and 3-fold GRAPPA acceleration, no significant changes in the strength of low frequency (<0.3 Hz) and high frequency connectivity (>0.35 Hz) in major RSNs was found, suggesting that the effects of spatial autocorrelations were similar despite considerable differences in g-factor related noise amplification. MB-EVI was considerably more sensitive than TR-matched dual-echo MB-EPI for mapping task-activation. Figure 13 shows an example of a motor-visual task comparing MB-EVI using multi-band factor 2 with a double-echo MB-EPI protocol using multi-band factor 8 in a patient with a low grade glioma. The average single voxel temporal SNR in supraventricular centrum semiovale in these scans was 29 and 26, respectively. MB-EVI provided larger volume coverage and displayed lower spurious false-positive activation than TR-matched double-echo MB-EPI at the same correlation threshold.

**Figure 14:**
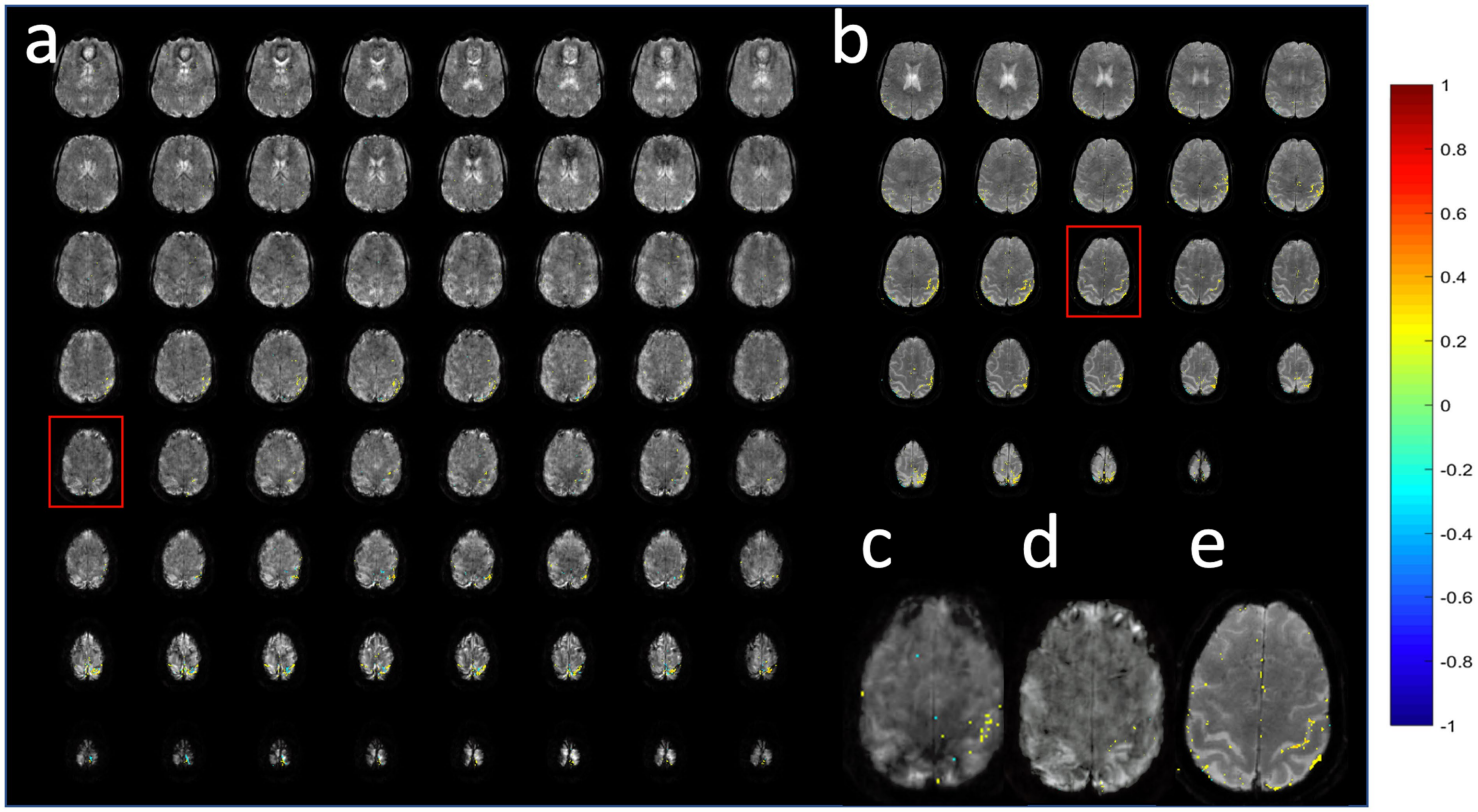
Comparison of **(a,c,d)** MB-EVI and **(b,e)** MB-EPI at 1 mm isotropic nominal voxel size in a healthy control during a finger-tapping task. Red boxes show slice locations of **(c–e)**. MB-EVI: TR/TE: 618/31 ms, multi-band factor: 4, flip angle: 25°, all 80 acquired slices are shown, numer of scans: 500 (5:09 min scan). **(c)** MB-EVI without T_2_* correction. **(a,d)** MB-EVI with T_2_* correction. MB-EPI: TR/TE: 932/30 ms, multi-band factor: 2, flip angle: 55°, 24 slices, slice gap: 1 mm, numer of scans: 500 (7:57 min scan). Images were processed with 1 mm isotropic Gaussian filter and correlation threshold: 0.3.

The introduction of k_z_-segmentation in the MB-EVI sequence on the Prisma scanner enabled a nominal 1 mm isotropic voxel size with large volume coverage at sub-second temporal resolution and sensitive mapping of task-based activation in short scan times (Figures 14a,c,d). MB-EVI reconstruction using the T_2_* correction (Figures 14a,d) increased the spatial resolution and image contrast, but reduced the SNR compared with the MB-EVI reconstruction without T_2_* correction (Figure 14c), thus reducing BOLD sensitivity. A MB-EPI acquisition with identical nominal voxel size and similar temporal resolution and volume coverage was only possible with much smaller number of slices and inter-slice gaps that were identical to the slice thickness (Figures 14b,e). Segmented MB-EVI enabled higher multi-band x GRAPPA acceleration with an acquisition rate of 8.77 ms/slice compared with MB-EPI at an acquisition rate of 38.46 ms/slice without aggravating multi-band artifacts, an increase of the acquisition speed of more than 4-fold compared with MB-EPI. MB-EVI enabled mapping of task-activation, despite locally increased sensitivity to B_0_-inhomogeneity and signal drifts. Figure 14 also illustrates the improved uniformity of the volume coverage of MB-EVI with reduced image intensity loss at the slab edges that was achieved with improved implementation of slab-selective RF pulses and the T_2_* correction compared with the initial implementation of MB-EVI on the Trio scanner.

Online reconstruction on the scanner and real-time image transfer to the external workstation were performed with time delays of less than one TR. The within-slab reconstruction of individual slices, slice reordering, slab concatenation, and the real-time fMRI analysis in TurboFIRE for isotropic voxel sizes as small as 2 mm was also performed with time delays of less than one TR. However, reconstruction performance decreased with isotropic voxel sizes smaller than 2 mm, resulting in increasing processing delays during the scan.

## Discussion

Combining multi-slab EVI with multi-band, i.e., simultaneous multi-slab, encoding rectifies primary inefficiencies of 2D encoding when many slices are needed as well as the temporal constraints of the slab-segmented EVI approach. Segmented k_z_ encoding provides flexibility in trading off acquisition speed vs. tolerance to B_0_-inhomogeneity, bridging the gap between single-shot 3D encoding (EVI) and traditional multi-shot 3D encoding (3D-EPI). It will thus be of interest to compare MB-EVI with efficiency-optimized 3D-EPI, which has been shown to exhibit higher sensitivity for mapping resting-state connectivity than TR-matched MB-EPI (Stirnberg et al., 2017), and with circular EPI (Rettenmeier et al., 2019). Our data show sensitive mapping of task activation and resting-state connectivity despite larger g-factors than with typical single- and dual-echo MB-EPI sequences. This tolerance to g-factor related noise amplification and the feasibility of using large multi-band acceleration factors in combination with large in-plane GRAPPA acceleration factors may in part due to the thickness of the slabs compared to the slice thickness in multi-band EPI, which results in a larger coil sensitivity variation between simultaneously excited slabs compared with simultaneously excited slices, enabling large overall acceleration factors. MB-EVI also provides flexibility for maximizing spatial and temporal resolution and volume-coverage depending on the choice of multi-band acceleration and in-plane GRAPPA acceleration, and it is compatible with 2D PF imaging. A distinct advantage of MB-EVI compared to other ultra-high temporal resolution fMRI appproach, such as for example MREG (Hennig et al., 2020) or Inverse Imaging (Lin et al., 2006), is the feasibility of online image reconstruction, which enables real-time fMRI analysis and online data quality control.

Direct comparisons of the fMRI sensitivity with MB-EPI are challenging due to the many parameters that determine spatial–temporal resolution and BOLD sensitivity and the observation that the spatial uniformity of slab excitation increases with decreasing TR and with increasing flip angle due to T_1_ saturation effects, necessitating low flip angles on the order of 10^o^ when using high accelerations factors and TRs of less than 300 ms. BOLD sensitivity at these short TRs was reduced when using the Ernst angle for excitation. This flip angle constraint limited the measured fMRI sensitivity gains at high temporal resolution despite comparable spatial SNR (Figure10). On the other hand, a moderately accelerated MB-EVI protocol at TR 400 ms provide considerably higher BOLD sensitivity and larger volume coverage than a dual-echo MB-EPI protocol with multi-band factor 8 at the same nominal voxel size and temporal resolution (Figure 13), which we have used in several clinical research studies in patients with brain tumors (Vakamudi et al., 2020). The short TR and high BOLD sensitivity of MB-EVI proved beneficial for mapping low and high frequency connectivity with higher spatial resolution and volume coverage (TR: 163 ms with 3 mm isotropic voxel size and 48 slices) compared with our previous study of high frequency connectivity using MS-EVI with TR: 136 ms, 4 mm isotropic voxel size and 16 slices (Trapp et al., 2018).

The motion sensitivity of MB-EVI manifests primarily at slab interfaces and can affect edge slices, whereas motion sensitivity within slabs is minor. This motion sensitivity of MB-EVI is mitigated by the higher temporal resolution of MB-EVI compared with MB-EPI.

Our results are in part consistent with the implementation of MB-EVI reported in (Chen and Feinberg, 2013), which demonstrated 3-fold multi-band acceleration for task-based fMRI at 3 mm isotropic spatial resolution using a checkerboard visual activation task. Comparable mean and maximum *t*-values in visual cortex were measured at TR: 480 ms for MB-EVI, MS-EVI, and MB-EPI with multi-band factor 8, and higher sensitivity was measured at TR: 480 ms compared with TR: 200 ms.

Further acceleration using 2D GRAPPA undersampling and reconstruction along the 2 phase encoding dimensions is expected to reduce g-factors compared with 1D GRAPPA used in the current study and enable larger number of slices per slab to reduce Fourier leakage. The implementation of this approach will be aided by full integration of the MB-EVI reconstruction into the manufacturer’s image reconstruction pipeline on the scanner instead of our current implementation that separately reconstructs the slice dimension on an external workstation. Our preliminary data also show the feasibility of retrospective compressed sensing with minor increases in spatial blurring and decreases of mean correlation coefficients on the order of 5% at acceleration factors up to 2.4. We thus expect that the integration of compressed sensing combined with parallel imaging (Lustig et al., 2007; Lustig et al., 2008) will enable further acceleration. This may enable MB-EVI with acquisition of multiple echo times in a single shot, exploiting sparsity in the TE domain. Multi-echo acquisitions not only increase BOLD sensitivity (Posse et al., 1999), but were also found to enable distinction of BOLD contrast-based resting state activity and of confounding physiological signal fluctuations, which can thus be automatically reduced (Wu et al., 2012; Kundu et al., 2012).

Significant improvements in image quality and reduction of signal dephasing are expected using improved shimming methods. Since the completion of this study, we have become aware that the manufacturer’s advanced shimming mode may not be optimal. Several recent studies have described improvement of single voxel and whole brain shimming using alternate shimming approaches (e.g., Vejdani Afkham and Alonso-Ortiz, 2025), which is consistent with our own recent experience using manual gradient shimming for spectroscopic imaging that achieved a significantly reduced spectral linewidth of the whole volume water signal compared to the advanced shimming mode.

NORDIC denoising, although not extensively tested in this study, was shown to be applicable to MB-EVI and particularly effective at reducing non-physiological signal fluctuations at high spatial resolution. Additional studies using data with different spatial and temporal resolutions and acceleration factors are needed to further characterize the performance of NORDIC for task-based and resting-state MB-EVI.

### Limitations

MB-EVI remains sensitive to B_0_-inhomogeneity due to the long EVI readout, despite k_z_-segmentation and k_z_ PF acquisition, which induces geometrical distortion and signal dephasing primarily in orbital frontal cortex and inferior temporal lobe. A possible solution at the expense of 2-fold reduction in temporal resolution is to acquire interleaved images with reversed k_z_-encoding gradients that counteract local gradients similar to Z-shimming, enabling efficient acquisition of fMRI scans with temporal resolution that is still superior that that of conventional multi-step Z-shimming. The current implementation of MB-EVI suffers from multi-band reconstruction related image nonuniformity and sensitivity loss at slab interfaces due to slab profile imperfections and T_1_ saturation. These can be addressed using nonlinear inversion for slab profile encoding (NPEN) (Wu et al., 2016). The sensitivity of MB-EVI to imperfections of slice profiles and movement related artifacts at slab edges will affect MB-EPI data as well, although in more subtle ways since they are not spatially resolved in MB-EPI data. Future research to mitigate these artifacts in MB-EVI may ultimately benefit MB-EPI as well.

Gains in spatial–temporal resolution were limited by increasing signal instability and ghosting when using larger than 4-fold multi-band acclerations, by signal instability in fMRI scans due to gradient instability and signal fluctuations due to respiration induced off-resonance effects. These can be addressed in part by adding navigators between k_z_encoding steps and by drift correction in the 2nd phase encoding direction.

In summary, combining MS-EVI with multi-band encoding enables high overall acceleration factors with up to 4-fold gains in spatial–temporal resolution compared with MB-EPI and provides flexibility for maximizing spatial–temporal resolution and volume coverage tailored to the neuroscience or clinical application. The high BOLD sensitivity of this hybrid MB-EVI approach and its compatibility with online image reconstruction enables high spatial–temporal resolution for real-time task-based and resting state fMRI.

## Data Availability

The raw data supporting the conclusions of this article will be made available by the authors, without undue reservation.

## Footnotes

^1^ https://github.com/SteenMoeller/NORDIC_Raw

^2^ http://www.mrc-cbu.cam.ac.uk/Imaging/mnispace.html

^3^ https://mialab.mrn.org
