# Corrigendum: Real-time fMRI using multi-band echo-volumar imaging with millimeter spatial resolution and sub-second temporal resolution at 3 tesla

**DOI:** 10.3389/fnins.2025.1612503

**Published:** 2025-05-01

**Authors:** Stefan Posse, Sudhir Ramanna, Steen Moeller, Kishore Vakamudi, Ricardo Otazo, Bruno Sa de La Rocque Guimaraes, Michael Mullen, Essa Yacoub

**Affiliations:** ^1^Department of Neurology, University of New Mexico, Albuquerque, NM, United States; ^2^Department of Physics and Astronomy, University of New Mexico, Albuquerque, NM, United States; ^3^Department of Radiology, Center for Magnetic Resonance Research, University of Minnesota, Minneapolis, MN, United States; ^4^Department of Medical Physics, Memorial Sloan Kettering Cancer Center, New York, NY, United States; ^5^Department of Radiology, Memorial Sloan Kettering Cancer Center, New York, NY, United States; ^6^Department of Nuclear Engineering, University of New Mexico, Albuquerque, NM, United States

**Keywords:** echo-volumar imaging, simultaneous multi slab encoding, multi-band encoding, functional MRI, resting-state connectivity, task-based activation, NORDIC denoising, compressed sensing

In the published article [Witzel et al., 2008 and Witzel et al., 2011] were not cited in the article and were missing from the reference list. The citations have now been inserted in **Introduction**, paragraph number two and should read:

“Recent advances in fMRI acquisition rates, using multi-band (simultaneous multi-slice) encoded EPI (MB-EPI) (Moeller et al., 2010; Setsompop et al., 2012), simultaneous image refocusing (Chen et al., 2015), magnetic resonance encephalography (MREG) (Hennig et al., 2020), highly accelerated echo-volumar imaging (Witzel et al., 2008, 2011) and multi-slab echo-volumar imaging (MS-EVI) (Posse et al., 2012), have played an important role in improving sensitivity for mapping functional connectomics (Feinberg et al., 2010; Smith et al., 2012; Posse et al., 2013) and for detecting task-based and resting state fMRI signal changes at frequencies above 0.2 Hz (Lewis et al., 2016).”

The following references have been added to the References list:

“Witzel, T., Polimeni, J. R., Wiggins, G. C., Lin, F., Biber, S., Hamm, M., et al. (2008). “Single-shot echo-volumar imaging using highly parallel detection,” in *Proceedings International Society of Magnetic Resonance in Medicine (ISMRM)* (Concord, CA: International Society for Magnetic Resonance in Medicine).”

“Witzel, T., Polimeni, J. R., Lin, F., Nummenmaa, A., Wald, L. L. (2011). “Single-shot whole brain echo volume imaging for temporally resolved physiological signals in fMRI,” in *Proceedings International Society of Magnetic Resonance in Medicine (ISMRM)* (Concord, CA: International Society for Magnetic Resonance in Medicine).”

In the published article, there was an error in the Funding statement. The acknowledgements for Lawrence L. Wald and Kawin Setsompop and their supporting grant application were erroneously excluded. The correct Funding statement appears below.

The author(s) declare that financial support was received for the research and/or publication of this article. Research reported in this publication was supported by NIH grant numbers 1R21EB022803, R21EB018494, 1R21CA241714, and R42NS134505-01. We gratefully acknowledge Lawrence L. Wald and Kawin Setsompop for sharing their expertise with highly accelerated EVI and for supporting grant application 1R21EB018494.

The authors apologize for this error and state that this does not change the scientific conclusions of the article in any way. The original article has been updated.

## References

[B1] Witzel T. Polimeni J. R. Wiggins G. C. Lin F. Biber S. Hamm M.. (2008). “Single-shot echo-volumar imaging using highly parallel detection,” in Proceedings International Society of Magnetic Resonance in Medicine (ISMRM) (Concord, CA: International Society for Magnetic Resonance in Medicine).

[B2] Witzel T. Polimeni J. R. Lin F. Nummenmaa A. Wald L. L. (2011). “Single-shot whole brain echo volume imaging for temporally resolved physiological signals in fMRI,” in Proceedings International Society of Magnetic Resonance in Medicine (ISMRM) (Concord, CA: International Society for Magnetic Resonance in Medicine).

